# Supplementary material for: Self-Assembly Nanoparticles of Natural Bioactive Abietane Diterpenes
Source: Int J Mol Sci. 2021 Sep 22;22(19):10210. doi: 10.3390/ijms221910210 (PMC8508833; doi:10.3390/ijms221910210)

International Journal of
Molecular Sciences

MDPI

Article

Self-assembly nanoparticles of natural bioactive abietane diterpenes

Epole Ntungwe [1,2], Eva María Domínguez-Martín [1,2], Gabrielle Bangay [1,2], Catarina Garcia, Eleonora Colombo[3], Andreia Rosatella[1,4], Lucilia Saraiva [5], Ana María Díaz-Lanza [2], Daniele Passarella[3] and Patrícia Rijo [1,4*]

1 CBIOS – Universidade Lusófona´s Research Center for Biosciences & Health Technologies, Campo Grande 376, 1749-024, Lisbon, Portugal; epole. ntungwe@ulusofona.pt; evam.dominguez@uah.es
2 University of Alcalá de Henares, Faculty of Pharmacy, Department of Biomedical Sciences, Pharmacology Area (Pharmacognosy Laboratory), New antitumor compounds: Toxic action on leukemia cells research group. Ctra. A2, Km 33.100 – Campus Universitario, 28805. Alcalá de Henares, Madrid, Spain; ana.diaz@uah.es
3 Dipartimento di Chimica, Università degli Studi di Milano, Via Golgi 19, 20133 Milano, Italy; eleonora.colombo@unimi.it
4 iMed.ULisboa, Faculdade de Farmácia da Universidade de Lisboa, Av. Prof. Gama Pinto, 1649-003 Lisboa, Portugal; andreia.rosatella@ulusofona.pt
5 LAQV - Faculty of Pharmacy of University of Porto, Rua de Jorge Viterbo Ferreira, 228 4050-313, Porto, Portugal; lucilia.saraiva@ff.up.pt

* Correspondence: patricia.rijo@ulusofona.pt

Supplementary Information

Figure S1: ^{1}H NMR for 12BzRoy-Sq (7)

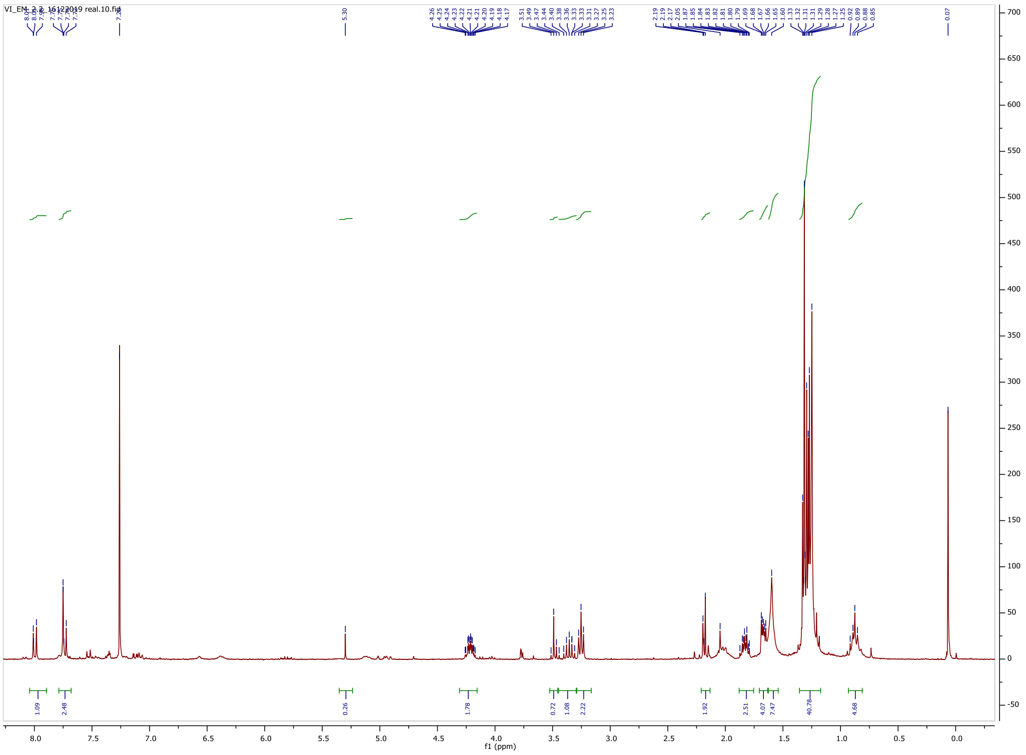

Figure S2: ^{1}H NMR for Roy-OA (5)

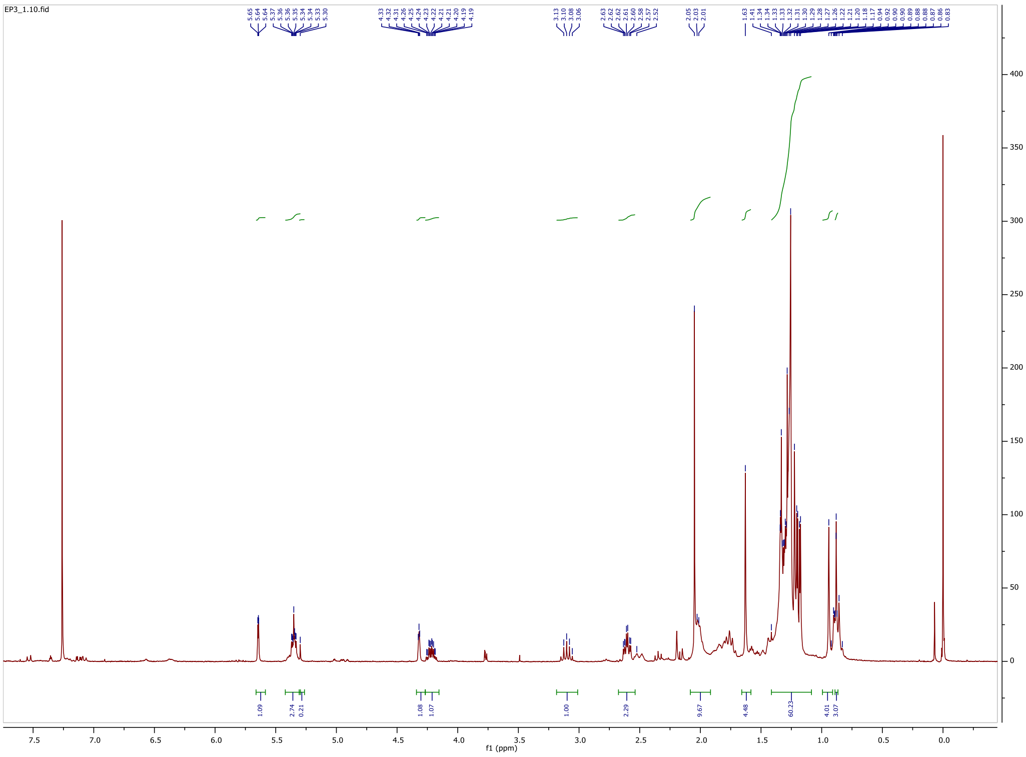

Supplement: Supplementary file 1 [file ijms-22-10210-s001.zip › ijms-1361616-supplementary.pdf]
